# Single-cell RNA sequencing and multiple bioinformatics methods to identify the immunity and ferroptosis-related biomarkers of SARS-CoV-2 infections to ischemic stroke

**DOI:** 10.18632/aging.204966

**Published:** 2023-08-21

**Authors:** Xiang Zhao, Qingyu Liang, Hao Li, Zhitao Jing, Dongmei Pei

**Affiliations:** 1 Department of Neurosurgery, The First Hospital of China Medical University, Shenyang, Liaoning 110001, China; 2 Department of Family Medicine, Shengjing Hospital, China Medical University, Shenyang, Liaoning 110001, China

**Keywords:** SARS-CoV-2, single-cell RNA sequencing, ischemic stroke, bioinformatic, ferroptosis

## Abstract

Background: Since December 2019, Coronavirus disease 2019 (COVID-19) induced by severe acute respiratory syndrome coronavirus 2 (SARS-CoV-2) has resulted in significant morbidity and mortality worldwide. There is an increased risk of ischemic stroke (IS) associated with COVID-19. However, few studies have been reported to explain the potential correlation between COVID-19 and IS.

Methods: We investigated the relationship and relevant mechanisms between COVID-19 and IS using single-cell RNA sequencing and multiple bioinformatics approaches.

Results: By intersecting differentially expressed genes and WGCNA critical module genes, we obtained 73 COVID-19-related IS genes. According to the KEGG pathway analysis, the COVID-19-related IS disease genes were significantly enriched in the hematopoietic cell lineage pathway, ribosome pathway, COVID-19 pathway and primary immunodeficiency pathway. Finally, three genes associated with immunity (B4GALT5, CRISPLD2, F5) and two genes associated with ferroptosis (ACSL1, CREB5) were identified up-regulated in COVID-19-related IS. Significantly, it was found that all five genes were highly expressed in monocytes by single cell RNA sequencing.

Conclusion: We believe these genes (B4GALT5, CRISPLD2, F5, ACSL1, CREB5) may regulate the immune response and ferroptosis of multiple immune cells, mainly including monocytes, which may contribute to the development of COVID-19-related IS. In addition, these genes may be potential targets for the treatment of COVID-19-related IS.

## INTRODUCTION

Coronavirus disease 2019 (COVID-19) induced by severe acute respiratory syndrome coronavirus 2 (SARS-CoV-2) has caused significant morbidity and mortality around the world since December 2019. COVID-19 is mainly a respiratory illness that may present as an acute upper and/or lower airway syndrome of various severity. The onset of symptoms of COVID-19 is more likely to occur gradually than the onset of flu. Patients may present with asymptomatic viral shedding or self-limiting symptoms such as fever, fatigue, myalgia, arthralgia, rhinorrhea, sore throat, and/or conjunctivitis [1]. But it can also develop into persistent fever, cough, hemoptysis, silent hypoxia, chest pain, respiratory failure, and even multi-organ failure [2]. Impaired sense of smell (hyposmia, anosmia and parosmia) or taste (dysgeusia) have been identified as important chemosensory disorders in COVID-19 [3]. Recently, a study has reported a potential association between COVID-19 and the development of idiopathic pulmonary fibrosis and chronic obstructive pulmonary disease in patients [4]. In addition, SARS-CoV-2 infection significantly increases mortality in patients with idiopathic pulmonary fibrosis [5]. A recent study reported that COVID-19 may increase the short-term incidence of ischemic stroke [6]. Other extrapulmonary manifestations include diarrhoea, lymphopenia, thrombocytopenia, impaired hepatic and renal function, rhabdomyolysis, meningoencephalitis, stroke, convulsions, cardiac arrhythmia or cardiac block, pancreatitis, Kawasaki disease such as multisystemic vasculitis, skin rash, thromboembolism, and acute thyroiditis [7]. Importantly, recent studies have shown COVID-19 increases the risk of ischemic stroke (IS) by approximately 5%, according to a World Stroke Organization panel review [8]. Ferroptosis is an iron-related form of programmed cell death [9]. It has been reported that ferroptosis plays an important role in COVID-19-induced brain injury [10, 11]. In 2012, ferroptosis was defined as an iron-dependent form of cancer cell death that differs from apoptosis, necrosis, and autophagy. This is a form of regulated cell death characterized by iron-dependent oxidative damage and subsequently ruptures of the plasma membrane and release of damage-associated molecules. To determine the sensitivity of ferroptosis, translational regulation of iron homeostasis is integrated with transcriptional regulation [12].

However, the relationship between COVID-19 and IS has been poorly studied. It is important to identify a common genetic signature between COVID-19 and IS which may provide useful guidance for the treatment of COVID-19-related IS. Here, using scRNA-seq and multiple bioinformatics methods, we have revealed the possible biological processes of COVID-19-related IS.

## MATERIALS AND METHODS

### Data download and processing

The single-cell dataset GSE165182 was obtained from the GEO (https://www.ncbi.nlm.nih.gov/geo/) database, which contained a total of 19 samples from patients with COVID-19 [13]. We downloaded the gene expression profile dataset GSE171110 containing 10 healthy tissues and 44 patients with COVID-19 [14]. A gene expression profile dataset GSE16561 containing 24 healthy tissues and 39 patients with IS was also downloaded [15]. Validation was conducted on the cohorts GSE157103, which included 26 normal samples, 100 COVID-19 patients, and GSE22255, which included 20 normal samples and 20 IS patients [16, 17]. We analyzed the quality of the single cell data set GSE165182 with the R-package Seurat after downloading the original data. We started by getting the annotation information of the probes, mapping them to the genes, removing multiple matches, and taking the median as the gene expression. Lastly, we obtained the gene expression profile.

### Single-cell quality control and dimensions reduction

We found cells expressing more than 200 genes, but fewer than 10,000 genes. Moreover, 20% of mitochondrial genes and 20% of ribosome genes were set as cut off values for further filtering. Using the “UMAP” diagram, we displayed and annotated cell clusters generated from 2000 hypervariable genes. All genes were scaled using the ScaleData function, and Principal Component Analysis (PCA) was performed. By using the “FindAllmarkers” function from Seurat R Package, we selected the ten genes with the highest expression levels in each cluster. In total, 30 clusters were discovered.

### Differential gene analysis and cell type annotation

Each cell subpopulation was identified using the FindAllMarkers function of the R package, and the gene expression profile data set was re-evaluated based on the CIBERSORT and ssGSEA [18] to identify the components of each subpopulation in the expression profile [19].

### Weighted co-expression network analysis in GSE148389

GSE171110 was analyzed using WGCNA, and we excluded outliers by selecting genes with SD >0. In addition to separating the data into different modules, we identified the modules most closely related to COVID-19 by setting the optimum soft threshold. Finally, to obtain more COVID-19-related genes after WGCNA analysis, we selected the blue and brown modules with the highest correlation for subsequent analysis.

### Differentially expressed genes (DEGs) identification

Differential analysis was performed on GSE171110 and GSE16561. After normalization, the “limma” R Package was used to examine the differences between the disease and normal sample groups. In order to obtain more differentially expressed genes, the value of logFC was set to 0.5. By applying a filter (logFC > 0.5 and adjusted *p* < 0.05), we finally obtained the intersection of the most significant module genes of WGCNA and DEGs.

### Functional and pathway enrichment analysis of intersect genes of COVID-19 and IS

A functional enrichment analysis was conducted on intersect genes. We selected the most enriched term as representative of the key terms based on their similarity of membership. Gene Ontology (GO) and Kyoto Encyclopedia of Genes and Genomes (KEGG) analyses were carried out using the ClusterProfiler R package (v4.0) to explore intersect genes’ functions and pathways. *P* < 0.05 was determined as the statistical significance.

### Machine learning for the diagnostic hub genes

In order to function the disease status predictions, two machine learning algorithms were employed. Using the “glmnet” R package, a least absolute shrinkage and selection operator (LASSO)-based algorithm was used to identify marker genes. In order to identify the set of genes with the highest discriminative power, support vector machine-recursive feature elimination (SVM-RFE) was applied using the “e1071” R package. And 9 marker genes have been acquired through the intersection genes between the two algorithms. The expression stages of 9 marker genes were further examined in the validation cohort. To investigate COVID-19-related pro-disease factors in IS, we identified up-regulated genes. Finally, three genes were selected from nine genes that were highly expressed in both COVID-19 and IS.

### Identification of key genes for ferroptosis in COVID-19-related IS

Ferroptosis-related genes were downloaded from FerrDb, [20] a web-based consortium providing a comprehensive and up-to-date database for ferroptosis markers, their regulatory molecules and associated diseases. A total of 728 ferroptosis-related genes were identified (Supplementary Table 1). We finally identified 2 ferroptosis genes associated with COVID-19-related IS.

### Immune infiltration analysis

CIBERSORT and ssGSEA were used to analyze the infiltration of hub genes, COVID-19, and normal samples. Bar plots were also used to visualize the percentages of each immune cell type in the samples and the relationship between COVID-19 and fifty significant immune pathways. The “pheatmap” package was used to create a heat map of immune cells, and the “vioplot” package was used to illustrate abundance. By using the “corrplot” package, we created a correlation heatmap to visualize the relationship between hub genes and different infiltrating immune cells.

### Statistical analysis

R was used for all statistical analyses. A student’s *t*-test was used to compare COVID-19, IS and normal samples. ROC analysis was conducted for the estimation of hub genes. Unless otherwise stated, statistical significance was set at *p* < 0.05.

### Data availability statement

The datasets generated in this study can be found in online repository. The names of the repository/repositories and accession number(s) can be found in the article/supplementary material.

## RESULTS

### Identification of DEGs in COVID-19 and IS

**Figure 1:**
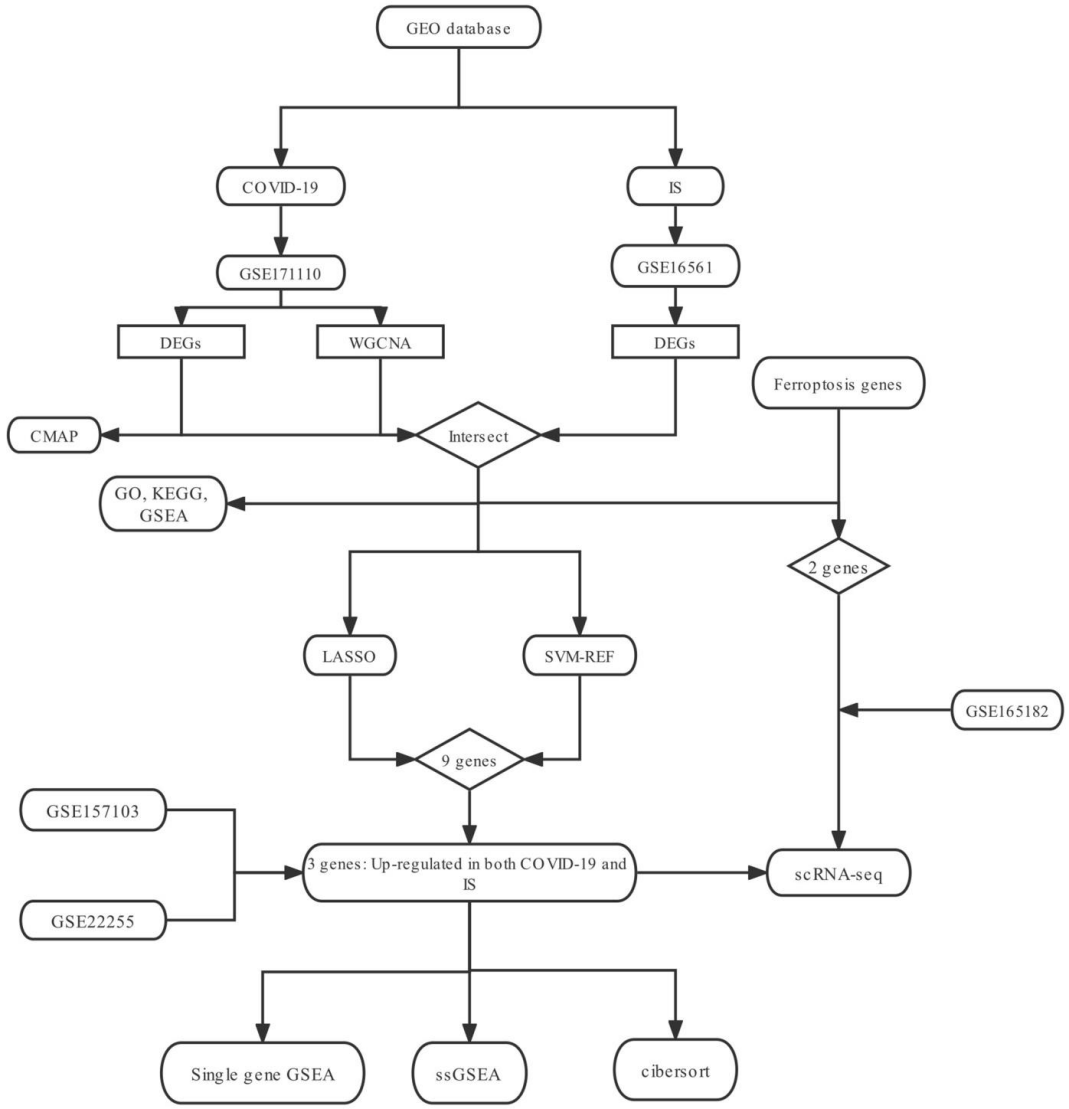
The research flow chart.

**Figure 2:**
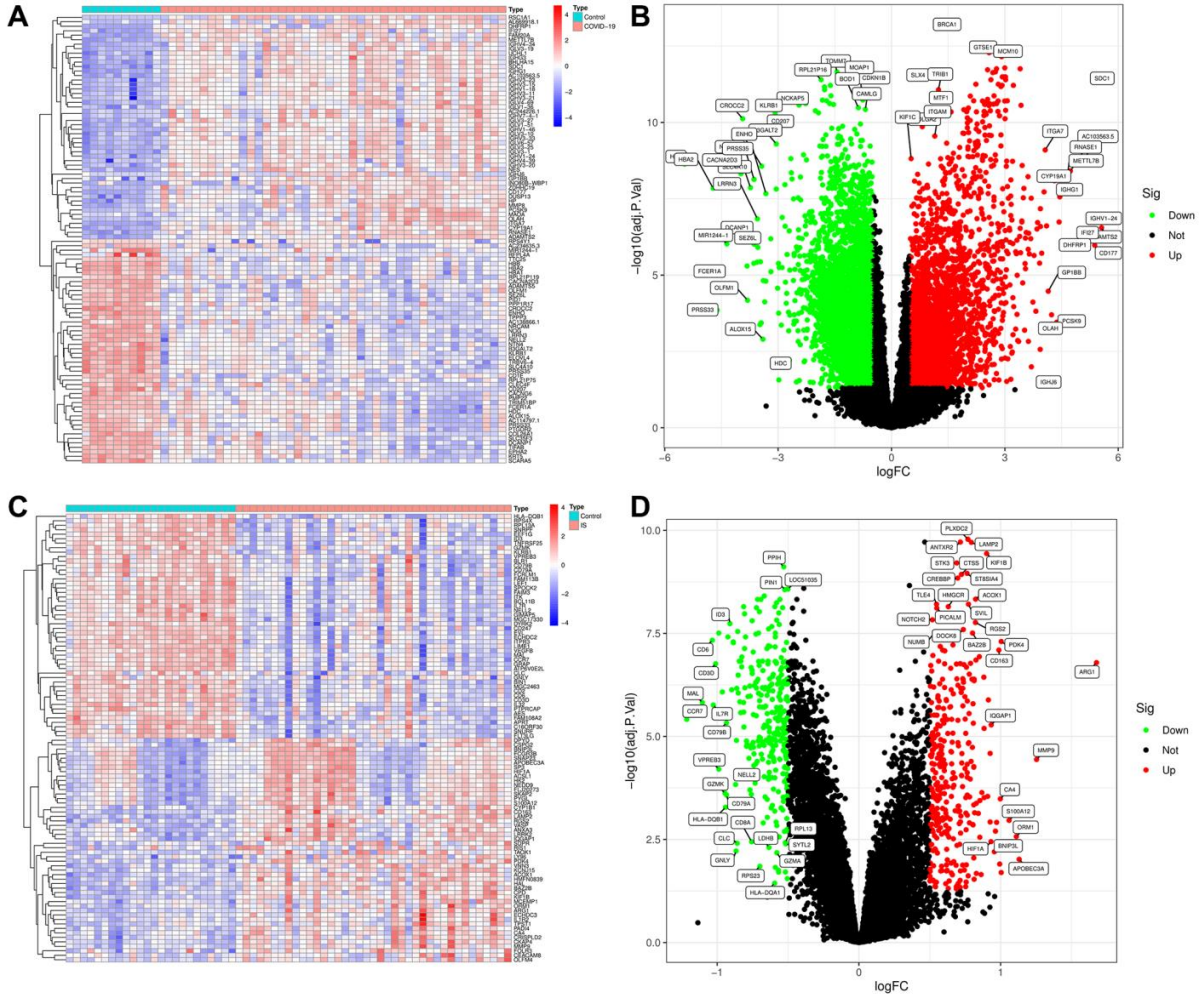
**Visualization and analysis of the differentially expressed genes (DEGs) in COVID-19 and IS.** (**A**) Heatmap clustering of genes with markedly different expression in COVID-19 compared with normal samples in GSE171110. (**B**) DEGs volcano map in COVID-19 compared with normal samples in GSE171110. (**C**) Heatmap clustering of genes with markedly different expression in IS compared with normal samples in GSE16561. (**D**) DEGs volcano map in IS compared with normal samples in GSE16561. |log2Foldchange|> 0.5 and adjusted *P*-value < 0.05 were used to define statistically significant DEGs.

To explore the biological mechanism of COVID-19-related IS, we conduct a comprehensive analysis based on GEO database (GSE171110. GSE16561) as showed in Figure 1. GEO dataset (GSE171110) contains 54 samples, including 10 normal samples and 44 COVID-19 samples. As a result of preprocessing and removing batch effects from the COVID-19 samples, 7047 DEGs were identified, including 3190 upregulated genes and 3857 downregulated genes (Supplementary Table 2), and heatmap (Figure 2A) and volcano map (Figure 2B) showed remarkable differences. There are 63 samples in the GEO dataset (GSE16561), including 24 normal samples and 39 IS samples. The heatmap (Figure 2C) and volcano map (Figure 2D) showed remarkable differences after the IS samples were preprocessed and batch effects removed.

### Weighted co-expression network analysis

**Figure 3:**
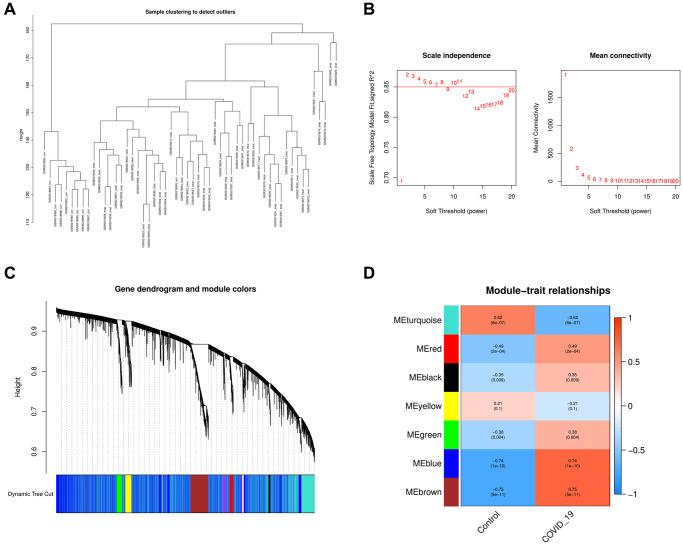
**Analysis of the weighted co-expression network in GSE171110.** (**A**) Sample clustering of dataset GSE171110. (**B**) The relationship between the scale-free fit index and various soft-thresholding powers; the relationship between the mean connectivity and various soft-thresholding powers. (**C**) Clustering dendrogram of genes, various colors represent different modules. (**D**) Analysis of correlations between modules and COVID-19. The blue module and brown module were significantly correlated with COVID-19 and normal samples.

As a first step, we identified 29302 genes with expression standard deviations greater than zero. We used the “flashClust” tool kit to perform cluster analysis with a threshold of 70 samples; cluster 2 contained 40 samples, which we kept (Figure 3A). To further filter out the entire power parameter range from 1 to 20, we set up a network with a soft threshold of b = 5 (R^2^ = 9) in the “WGCNA” package (Figure 3B). The threshold for merging similar module groups is 0.25; the minimum number of modules is 50. Genes that co-expressed with each other were produced in seven modules (Figure 3C). These findings suggested that multiple modules were associated with COVID-19. The blue and brown modules were the most significant, containing 5096 genes (Figure 3D).

### Identification of COVID-19-related IS genes and functional enrichment analysis

**Figure 4:**
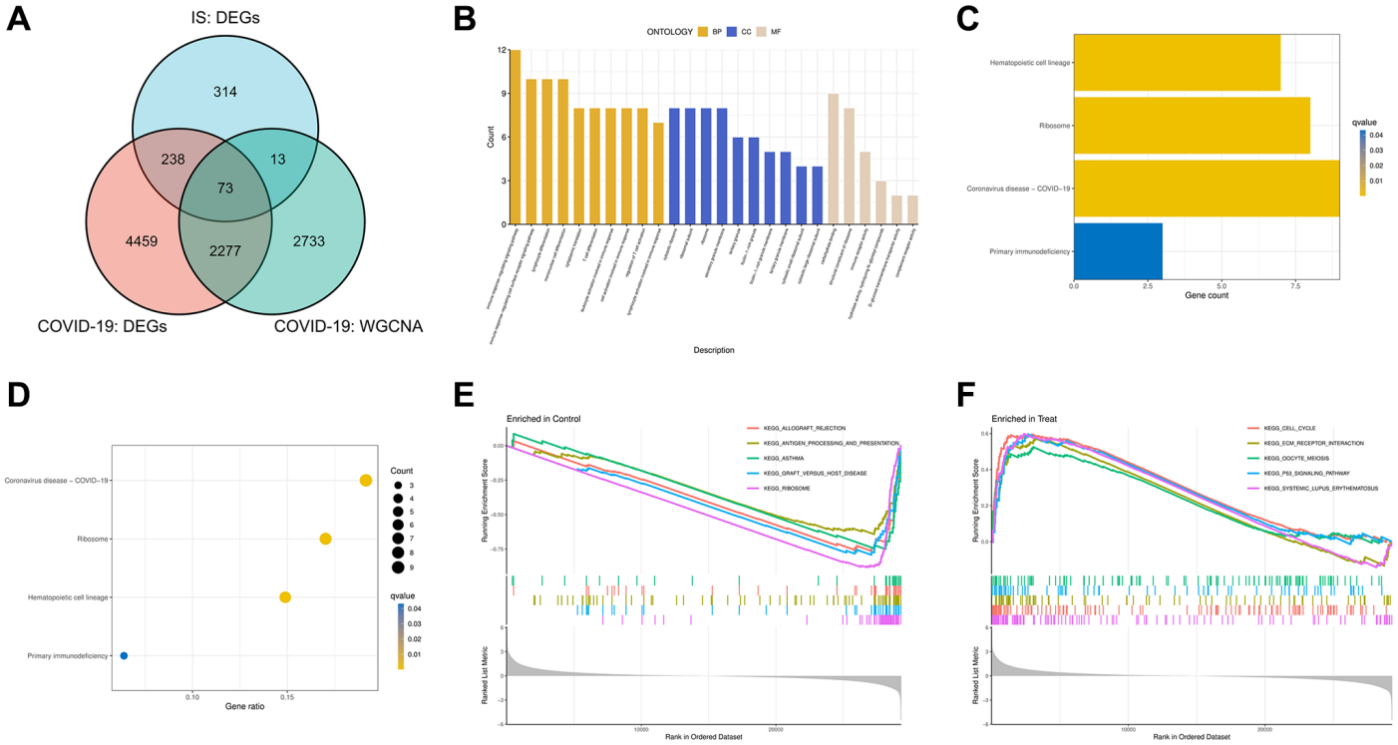
**Intersect genes and functional enrichment analysis.** (**A**) Venn diagram of the intersect genes of DEGs of COVID-19, IS and WGCNA hub genes of COVID-19. (**B**) Gene Ontology (GO) analyses for intersect genes. (**C**, **D**) Kyoto Encyclopedia of Genes and Genomes (KEGG) analysis of intersect genes. (**E**, **F**) Gene set enrichment analyses (GSEA) of GSE171110.

By intersecting DEGs and WGCNA critical module genes, we obtained 73 COVID-19-related IS genes (Figure 4A). In order to investigate the potential biological functions of COVID-19-related IS genes, GO and KEGG pathway functional enrichment analyses were conducted. The GO results revealed that the BP primarily associated with the immune response, lymphocyte differentiation, mononuclear cell differentiation, cytoplasmic translation and regulation of T cells. For CC enrichment analysis, the results showed that COVID-19-related IS disease genes significantly took part in ribosome and secretory granule membrane. In the enrichment analysis of MF, the COVID-19-related IS disease genes mainly revolved in carbohydrate binding, structural constituent of ribosome, and immune receptor activity (Figure 4B). According to the KEGG pathway analysis, the COVID-19-related IS disease genes were significantly enriched in the hematopoietic cell lineage pathway, ribosome pathway, COVID-19 pathway and primary immunodeficiency pathway (Figure 4C, 4D). Finally, we also constructed GSEA analysis, and the results showed cell cycle, ECM receptor interaction, oocyte meiosis, P53 signaling pathway and system lupus erythematosus were enriched in the COVID-19 group (Figure 4E), while allograft rejection, antigen processing and presentation, asthma, graft versus host disease and ribosome were enriched in the control group (Figure 4F).

### Identification of hub genes in COVID-19-related IS

**Figure 5:**
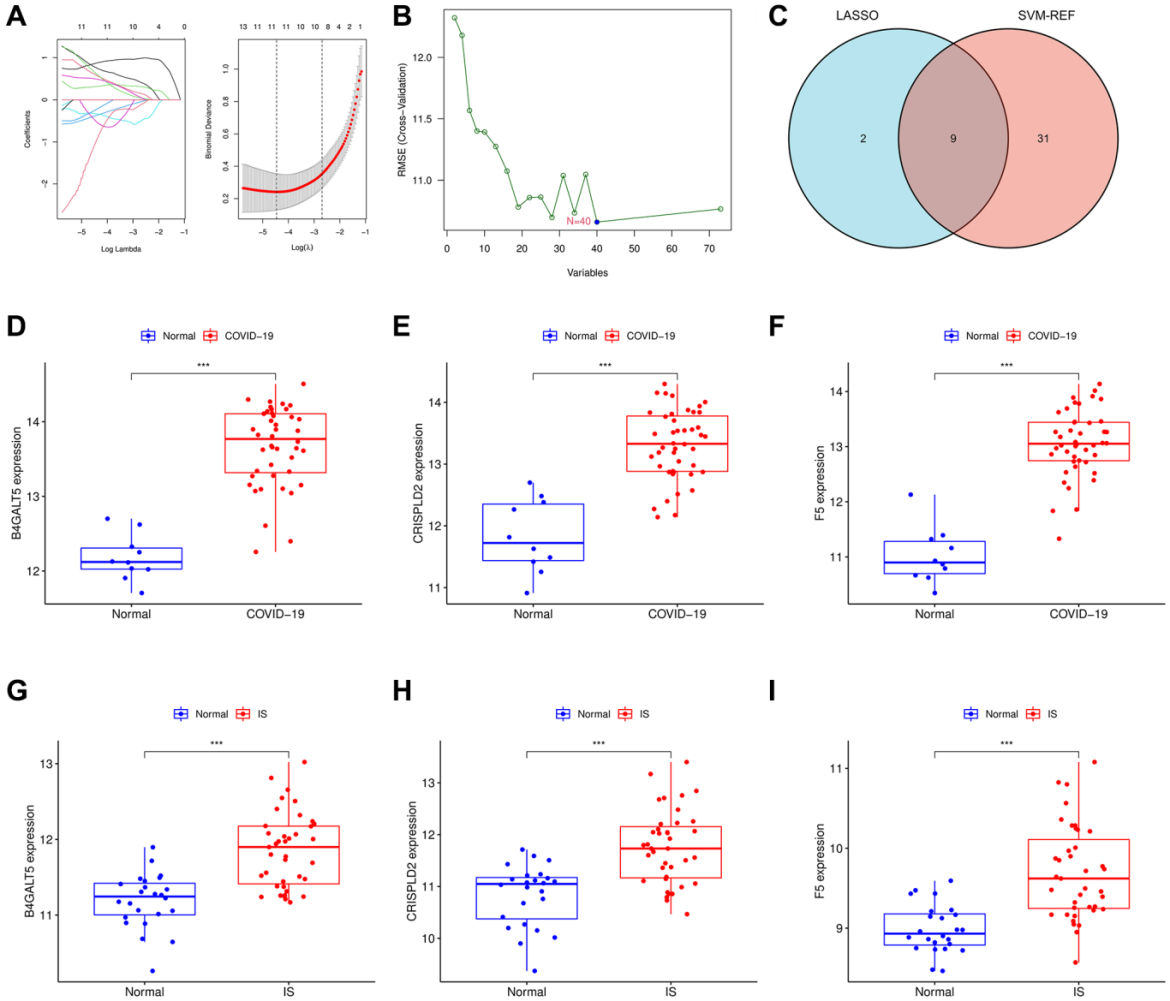
**Identification of the hub genes using machinery methods.** (**A**) Fine-tuning the least absolute shrinkage and selection operator (LASSO) model’s feature selection. LASSO regression was used to narrow down the DEGs, resulting in the discovery of 11 variables as potential markers. The ordinate represents the value of the coefficient, the lower abscissa represents log (λ), and the upper abscissa represents the current number of non-zero coefficients in the model. (**B**) A plot illustrating the process of selecting biomarkers using the support vector machine-recursive feature elimination (SVM-RFE) technique. The SVM-RFE technique was used to identify a subset of 40 characteristics. (**C**) Intersection LASSO and SVM-RFE analysis was displayed in a Venn diagram. B4GALT5, CRISPLD2 and F5 were chosen as hub genes which up-regulated in both COVID-19 and IS. (**D**–**F**) B4GALT5, CRISPLD2 and F5 mRNA expression in COVID-19 compared to normal samples in GSE171110. (**G**–**I**) B4GALT5, CRISPLD2 and F5 mRNA expression in IS compared to normal samples in GSE16561.

In order to identify potential biomarkers of COVID-19-related IS, two bioinformatic algorithms were used. Based on LASSO regression, 11 intersect genes were identified as diagnostic biomarkers for COVID-19-related IS (Figure 5A). SVM-RFE was used to identify a subset of 40 genes (Figure 5B). We finally selected the 9 overlapping characteristic genes (B4GALT5, CRIP2, CRISPLD2, F5, ITM2C, OGFRL1, RPL4, RPL13A, RPL22) using LASSO and SVM-RFE algorithm (Figure 5C). We then analyzed the mRNA expression of 9 genes (Supplementary Figures 1, 2), and 3 genes (B4GALT5, CRISPLD2, F5) were found up-regulated in both COVID-19 and IS (*p* < 0.001, Figure 5D–5I). In the validation cohort GSE157103 and GSE22255, the levels of the three biomarkers (B4GALT5, CRISPLD2, F5) have been further investigated to produce more precise and reproducible results, which were found to be significantly up-regulated in disease group than control group, (*p* < 0.001; Supplementary Figure 3).

### Diagnostic effectiveness and single gene ssGSEA analysis of the hub genes

**Figure 6:**
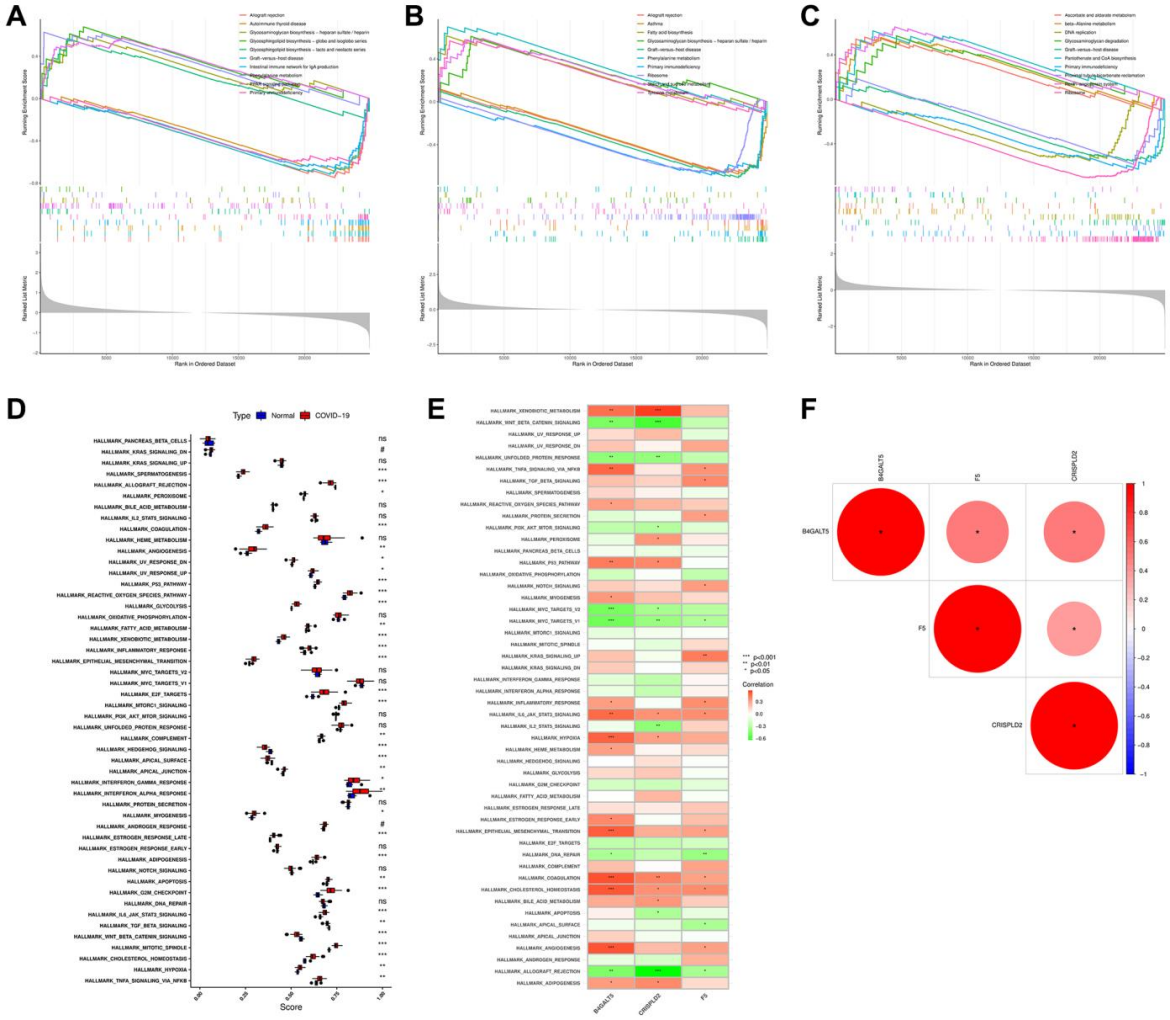
**Gene set enrichment analyses (GSEA) analysis of 3 hub genes and the relationship between COVID-19 and 50 significant pathways.** (**A**) Gene set enrichment analyses (GSEA) analysis of B4GALT5. (**B**) Gene set enrichment analyses (GSEA) analysis of CRISPLD2. (**C**) Gene set enrichment analyses (GSEA) analysis of F5. (**D**) Analysis of the relationship between COVID-19 and 50 significant pathways. (**E**) Analysis of the relationship between 3 hub genes and 50 significant pathways. (**F**) Correlation heat map showing the correlation between 3 hub genes.

Using the identified genes, a logistic regression algorithm was used to construct the diagnosis model. In addition, we used AUC to quantify the capability of discrimination. As shown in Supplementary Figure 4, characteristic biomarkers showed high diagnostic efficacy in distinguishing COVID-19-related IS from control samples, with an AUC of 0.984 (95% CI 0.950–1,000) for B4GALT5, 0.966 (95% CI 0.914–0.998) for CRISPLD2, and 0.991 (95% CI 0.966 to 1.000) for F5. This further strengthens the diagnostic capability of these three characteristic genes as potential biomarkers for the diagnosis of COVID-19-related IS. Single gene analysis of ssGSEA was performed, and the results showed glycosphingolipid biosynthesis, phenylalanine metabolism, and PPAR signaling pathway were enriched in B4GALT5 high expression samples, while allograft rejection, autoimmune thyroid disease, graft–versus–host disease, intestinal immune network and primary immunodeficiency were enriched in B4GALT5 low expression samples (Figure 6A). As for CRISPLD2, the high expression group contained fatty acid biosynthesis, glycosaminoglycan biosynthesis, phenylalanine metabolism, starch and sucrose metabolism and tyrosine metabolism pathway, while low expression group contained allograft rejection, graft–versus–host disease, primary immunodeficiency, asthma and ribosome pathway (Figure 6B). High F5 expression group was involved in ascorbate and aldarate metabolism, beta–Alanine metabolism, glycosaminoglycan degradation, pantothenate and CoA biosynthesis, renin–angiotensin system pathway, while low F5 expression group was involved in DNA replication, graft–versus–host disease, primary immunodeficiency, proximal tubule bicarbonate reclamation and ribosome pathway (Figure 6C). In addition, we analyzed the relationship of 50 signature functional pathways with COVID-19 and hub genes using ssGSEA. The results showed many biological functional processes were closely related to the occurrence of COVID-19 including spermatogenesis, allograft rejection, peroxisome, coagulation, angiogenesis, p53 pathway, reactive oxygen species pathway, glycolysis, fatty acid metabolism, xenobiotic metabolism, inflammatory response, epithelial mesenchymal transition, E2F targets, mtorc1 signaling, complement, hedgehog signaling, apical, interferon response, myogenesis, estrogen response late, adipogenesis, apoptosis, g2m checkpoint, il6 jak stat3 signaling, TGF beta signaling, WNT beta catenin signaling, mitotic spindle, cholesterol homeostasis, hypoxia and TNFA signaling via NFKB (*p* < 0.05, Figure 6D). Importantly, as shown in Figure 6E, B4GALT5 was found to have a positive correlation with xenobiotic metabolism, TNFA signaling via NFKB, reactive oxygen species pathway, p53 pathway, myogenesis, inflammatory response, IL6 JAK STAT3 signaling, hypoxia, heme metabolism, estrogen response early, epithelial mesenchymal transition, coagulation, cholesterol homeostasis, angiogenesis and adipogenesis (*p* < 0.05), and a negative correlation with WNT beta catenin signaling, unfolded protein response, MYC targets, DNA repair and allograft rejection (*p* < 0.05). CRISPLD2 was found to have a positive correlation with xenobiotic metabolism, peroxisome, p53 pathway, IL6 JAK STAT3 signaling, hypoxia, coagulation, cholesterol homeostasis, bile acid metabolism and adipogenesis (*p* < 0.05), while a negative correlation with WNT beta catenin signaling, unfolded protein response, PI3k AKT mTOR signaling, MYC targets, IL2 STAT5 signaling, apoptosis and allograft rejection (*p* < 0.05). F5 was found to have a positive correlation with TNFA signaling via NFKB, TGF beta signaling, protein secretion, notch signaling, KRAS signaling up, inflammatory response, IL6 JAK STAT3 signaling, epithelial mesenchymal transition, coagulation, cholesterol homeostasis and angiogenesis (*p* < 0.05), and a negative correlation with DNA repair, apical surface and allograft rejection (*p* < 0.05). Finally, gene correlation analysis indicated that the three genes were positively correlated with each other (*p* < 0.05, Figure 6F).

### Immune cell infiltration

**Figure 7:**
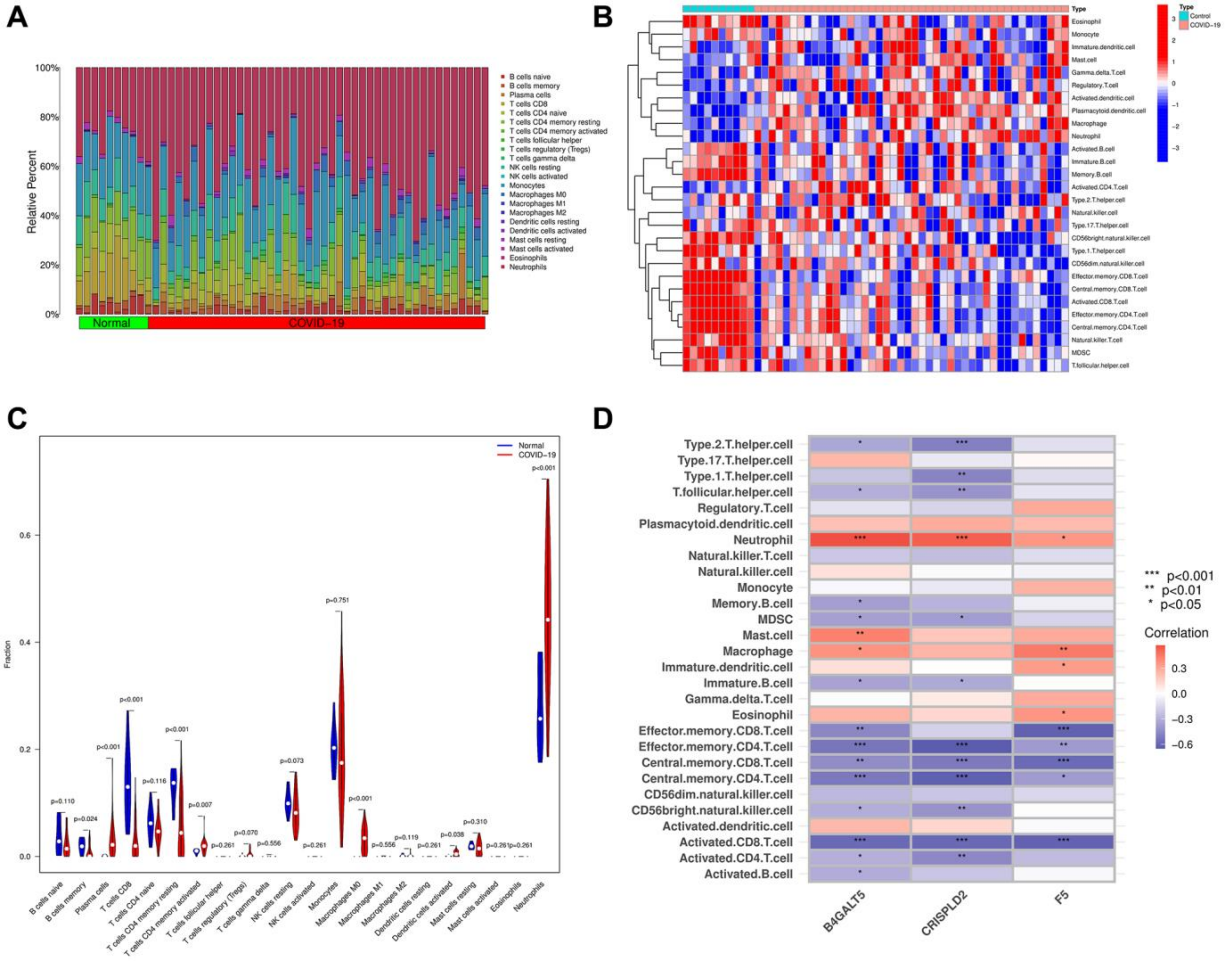
**The composition of immune cells was analyzed and displayed.** (**A**) Heat map of the 22 immune cell subpopulations in GSE171110 using CIBERSORT. (**B**) Heat map of immune cell infiltration in GSE171110 using ssGSEA. (**C**) Violin diagram illustrating the proportion of different kinds of immune cells in COVID-19 and normal samples using ssGSEA. (**D**) Correlation heat map showing the correlation between 28 different kinds of immune cells and 3 hub genes. The stronger the connection, the redder the hue. (*P*-values < 0.05 were considered as statistically significant. ^*^P < 0.05; ^**^P < 0.01; ^***^P < 0.001. Red indicates a positive correlation, while blue indicates a negative correlation).

First, we summarized the results from 10 normal and 44 COVID-19 samples using the CIBERSORT algorithm (Figure 7A). In addition, we used ssGSEA algorithm to analyze immune cells subtypes between COVID-19 samples and normal samples (Figure 7B). As illustrated in Figure 7C, COVID-19 patients showed high infiltration of plasma cells, CD4^+^ T cells memory activated, M0 macrophages, dendritic cells activated and neutrophils than normal samples, while normal patients showed high level of B cells memory, CD8^+^ T cells and CD4^+^ T cells memory resting than COVID-19 patients. We also analyzed the relationship between the 3 hub genes and immune cells subtypes via a correlation heatmap, which indicated B4GALT5 had strong positive correlation with neutrophils, mast cells and Macrophages (*p* < 0.05), and had negative correlation with Th follicular cells, Th2 cells, memory B cells, MDSC, immature B cells, effector memory CD8^+^ T cells, effector memory CD4^+^ T cells, central memory CD8^+^ T cells, central memory CD4^+^ T cells, CD56^-^ natural killer cells, activated CD8^+^ T cells, activated CD4^+^ T cells and activated B cells (*p* < 0.05). CRISPLD2 had strong positive correlation with neutrophil, and had negative correlation with Th1 cells, Th2 cells, Th follicular cells, MDSC, immature B cells, effector memory CD4^+^ T cells, central memory CD8^+^ T cells, central memory CD4^+^ T cells, CD56^-^ natural killer cells, activated CD8^+^ T cells and activated CD4^+^ T cells (*p* < 0.05). F5 had strong positive correlation with neutrophils, macrophages, immature dendritic cells and eosinophils (*p* < 0.05), and had negative correlation with effector memory CD8^+^ T cells, effector memory CD4^+^ T cells, central memory CD8^+^ T cells, central memory CD4^+^ T cells and activated CD8^+^ T cells (*p* < 0.05, Figure 7D).

### Definition of clusters and dimensionality reduction analysis

**Figure 8:**
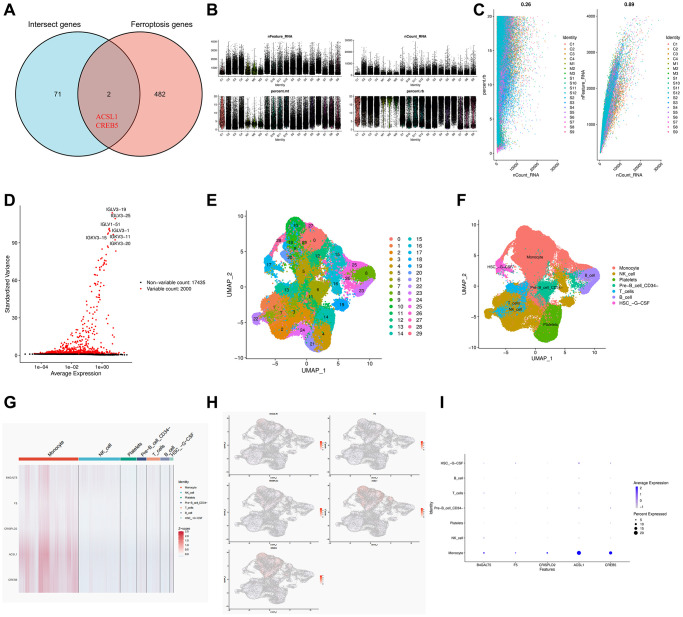
**Single cell RNA sequencing in COVID-19 based on GSE165182.** (**A**) Venn diagram of the intersect genes of intersect genes and ferroptosis genes. (**B**, **C**) The proportion of mitochondrial and ribosome genes is adjusted to ensure the quality of cell samples. (**D**) 2000 highly variable genes are indicated in red, with the 10 most important emphasized. (**E**, **F**) Reduced dimensionality and cluster analysis using UMAP. (**G**) Heat map showing the 5 hub genes in different cell types annotated by scRNA-seq. (**H**) Distribution of 5 hub genes in cell clusters. (**I**) Bubble diagram showing the distribution of the 5 hub genes in different cell types.

The single cell data set was subjected to quality control procedures. In Figure 8B, 8C, we removed some cells and controlled mitochondrial and ribosomal genes to ensure the quality of the samples. Then we identified 2000 highly variable genes and labeled the top 10. Hypervariable genes are highlighted in red in Figure 8D. Using UMAP, the cells are grouped into 30 clusters, which can be classified into monocytes, NK cells, platelets, pre-B cells, CD34^+^ T cells, B cells, and HSC–G–CSF (Figure 8E, 8F).

### Ferroptosis-related genes and hub genes-related scRNA-seq

The possible potential role of ferroptosis mechanism between COVID-19 and IS was considered. As a result of intersecting 73 COVID-19-related IS genes with ferroptosis genes, we obtained two ferroptosis genes (ACSL1, CREB5) that were relevant to COVID-19-related IS (Figure 8A). Next, we analyzed the distribution of these two ferroptosis genes and three previous hub genes at the cellular level in COVID-19 patients using scRNA-seq. The heat map displayed the distribution of the five genes in different cell types (Figure 8G). As shown in Figure 8H, 8I, B4GALT5Z was mainly distributed in monocytes, NK cells, T cells and HSC-G-CSF cells. F5 was mainly distributed in monocytes, T cells and HSC-G-CSF cells. CRISPLD2 was mainly distributed in monocytes. While the ferroptosis gene ACSL1 and CREB5 were mainly distributed in monocytes, HSC-G-CSF cells and pre-B CD34^−^ cells. Importantly, all five genes were highly expressed in monocytes from COVID-19 patients.

### Drug prediction for COVID-19

We queried the CMAP database with DEGs from the filtered COVID-19 gene expression profile. The compounds producing expression changes which were the reverse of those seen in our COVID-19 profile are shown in Supplementary Table 3. Among the drugs with the lowest connectivity scores, ten compounds were selected from the list using the criteria previously described. These compounds included inhibitors of EGFR, MEK, topoisomerase, HDAC, tubulin and CDK (Supplementary Figure 5). Finally, we used a schematic diagram to illustrate the research (Supplementary Figure 6).

## DISCUSSION

It is vital to study the complications associated with COVID-19. A growing number of studies have reported a strong association between COVID-19 and the nervous system [21, 22]. One study has even reported that COVID-19 causes changes in the cerebrospinal fluid of patients [23]. However, there is little research on the relationship between COVID-19 and IS. In this study, an innovative approach was developed to combine scRNA-seq analysis with a variety of bioinformatic approaches to determine the potential mechanisms in COVID-19-related IS.

We first identified 73 critical genes of COVID-19-related IS (Figure 4A). In addition, functional enrichment analysis results showed that these genes of COVID-19-related IS were mainly enriched in immune-related pathways (Figure 4B–4F). Then we screened 3 hub genes (B4GALT5, CRISPLD2, F5) which were up-regulated in both COVID-19 and IS by multiple machine learning methods combined with the mRNA expression of genes (Figure 5). B4GALT5, also known as galactosyltransferase V, is one of seven members of the -1,4 galactosyltransferase family. The B4GALT glycosyltransferase has been extensively studied in recent years. During glycoconjugate synthesis, these enzymes shift galactosyl groups from UDP galactosides to N-acetylglucosamine or another glycosyl acceptor. Studies have shown that 1, 4 galactosyltransferases play a role in embryonic development, neurological development, immune and inflammatory responses, tumor formation, and many other life processes [24]. However, the role of B4GALT5 in COVID-19 and IS was not known. There has been evidence that CRISPLD2 plays a role in anti-inflammatory responses and tissue remodeling. There has been evidence that CRISPLD2 plays a role in anti-inflammatory responses and tissue remodeling [25]. LCCL domain-containing cysteine-rich secretory protein containing 2 (CRISPLD2), a novel LPS-binding protein, binds directly to LPS in bloodstream and inhibits its binding to its original target receptor, TLR4, reducing inflammation caused by LPS. A fascinating aspect of the study is that LPS itself can induce the release of CRISPLD2 *in vitro* and *in vivo* via TLR4-mediated signaling. Furthermore, immunological cells could be enhanced to secrete CRISPLD2 when they had access to an anti-hTLR4 specific antibody (anti-hTLR4-IgAmA), which had been used to block LPS-induced inflammation [26]. Coagulation factor V (F5) is a high-molecular-weight procofactor (330 kDa) that plays a critical role in blood coagulation. By activating coagulation factor X, it converts prothrombin to thrombin as a cofactor [27]. Here, a positive correlation was found between B4GALT5 and xenobiotic metabolism, TNFA signaling via NFKB, reactive oxygen species, p53 signaling, myogenesis, inflammation, IL6 JAK STAT3 signaling, hypoxia, heme metabolism, estrogen response early, epithelial mesenchymal transition, coagulation, cholesterol homeostasis, angiogenesis and adipogenesis (*p* < 0.05). A positive correlation was found between CRISPLD2 and xenobiotic metabolism, peroxisomes, the p53 pathway, IL6 JAK STAT3 signaling, hypoxia, coagulation, cholesterol homeostasis, bile acid metabolism, and adipogenesis (*p* 0.05). TNFA signaling via NFKB, TGF beta signaling, protein secretion, notch signaling, KRAS signaling up, inflammatory response, IL6 JAK STAT3 signaling, epithelial mesenchymal transition, coagulation, cholesterol homeostasis and angiogenesis were found to be positively correlated with F5 (*p* < 0.05). Furthermore, gene correlation analysis revealed that the three genes were positively correlated (*p* < 0.05, Figure 6). The results of immune cell infiltration analysis showed that three genes are closely related to multiple immune cells (*p* < 0.05, Figure 7). Therefore, B4GALT5, CRISPLD2 and F5 may be potent targets for the treatment of COVID-19-associated IS.

Importantly, we also obtained 2 ferroptosis genes (ACSL1, CREB5) of COVID19-related IS. And we found seven major cell types, such as monocytes, NK cells, platelets, CD34^−^ pre-B cells, T cells, B cells and HSC-G-CSF in patients with COVID19. The majority of B4GALT5Z was found in monocytes, NK cells, T cells, and HSC-G-CSF cells. There was a high concentration of F5 in monocytes, T cells, and HSC-G-CSF cells. In monocytes, CRISPLD2 was mainly found. The ferroptosis genes ACSL1 and CREB5 were mainly present in monocytes, HSC-G-CSF cells, and pre-B CD34^−^ cells. The monocytes of COVID-19 patients showed high levels of expression of all five genes (Figure 8). As a result of excessive lipid peroxidation and subsequent cell membrane damage, ferroptosis is a newly identified iron-dependent necrotic cell death [28]. As a result of esterification by acyl-CoA synthetase long-chain member 4 (ACSL4), polyunsaturated fatty acids (PUFAs) can rupture the cell membrane at the execution stage [29]. ACSL1 has recently been identified as a ferroptosis promoter [30]. Ferroptosis was induced by ACSL1-induced eleostearic acid (ESA). In contrast, ESA-triggered, ACSL1-dependent ferroptosis differed significantly from that induced by GPX4 inhibitor ML160 and FSP1 inhibitor iFSP1 inhibitors, which are canonical inducers of ferroptosis [31]. Recently, a study indicated that ferroptosis inhibition can protect hosts from MHV-A59 infection. Targeting ferroptosis may serve as a potential treatment approach for dealing with hyper-inflammation induced by coronavirus infection [32]. As for CREB5, gene CREB5 encodes a transcription activator found in eukaryotic cells and is located on chromosome 7 (7p15.1). As a member of the ATF/CREB family, CREB5 has a high affinity for cAMP-responsive elements (CREs). There are a number of genes in the ATF/CREB family that are targeted, including transcriptional regulators (chromatin-modifying enzymes, coactivators, and corepressors), mitochondrial homeostasis genes, protein import genes, proteases, transporters, chaperones, metabolism, and cell cycle entry. In addition to containing zinc finger domains at its N-terminus and a leucine zipper domain at its C-terminus, the CREB5 protein is an ATF/CREB family member. An essential transcription factor, CREB5 functions as a CRE-dependent trans-activator in a homodimer or heterodimer with c-Jun and CRE-BP1 [33–36]. Significantly, a research revealed CREB5 gene was highly expressed in SARS-CoV-2 infected patients and was associated with prognosis in patients with coronavirus infection, which was consistent with the results of our study [37]. During inflammation, monocytes are heterogeneous cells that circulate in the blood and play a key role in the innate immune response. Depending on the local cytokine environment, these cells can be differentiated into macrophages or dendritic cells (DCS) after transporting antigens to lymph nodes and aggregating at sites of inflammation [38]. Some studies have reported that monocytes play an important role in the COVID-19 process [39, 40]. Study findings indicate that COVID-19 viral load declines with NK cell status, and NK cells are capable of controlling SARS-CoV-2 replication by recognizing infected cells [41]. In addition, several studies have confirmed that B and T cells can mediate COVID-19 immunity [42, 43]. However, it remains unclear why CD34^−^ pre-B cells and HSC-G-CSF are related to COVID-19 and IS.

In our study, five disease marker genes were all confirmed to be highly expressed in monocytes, and these five genes could be potential targets for the treatment of COVID-19-related IS.

### Limitations and future directions

This study was limited by the clinical case samples and the limited depth to explore the specific association mechanism between IS and COVID-19. In the future, we hope to collect more COVID-19-related IS samples and explore the potential association between these two diseases in a deeper level.

## CONCLUSIONS

In summary, this study explored the potential correlation between COVID-19 and IS using single-cell RNA sequencing and multiple bioinformatics methods. We identified three immune-related genes (B4GALT5, CRISPLD2, F5) and two ferroptosis genes (ACSL1, CREB5), all five genes were highly expressed in monocytes. Our results suggested that these genes may be involved in the development of COVID-19-related IS by regulating the immune response and ferroptosis of multiple immune cells, mainly including monocytes. Importantly, these genes may be potential targets for the treatment of COVID-19-related IS.

## Supplementary Materials

Supplementary Figures

Supplementary Tables

## References

[1] To KK, Sridhar S, Chiu KH, Hung DL, Li X, Hung IF, Tam AR, Chung TW, Chan JF, Zhang AJ, Cheng VC, Yuen KY. Lessons learned 1 year after SARS-CoV-2 emergence leading to COVID-19 pandemic. Emerg Microbes Infect. 2021; 10:507–35. 10.1080/22221751.2021.1898291 33666147 PMC8006950

[2] Docherty AB, Harrison EM, Green CA, Hardwick HE, Pius R, Norman L, Holden KA, Read JM, Dondelinger F, Carson G, Merson L, Lee J, Plotkin D, et al, and ISARIC4C investigators. Features of 20 133 UK patients in hospital with covid-19 using the ISARIC WHO Clinical Characterisation Protocol: prospective observational cohort study. BMJ. 2020; 369:m1985. 10.1136/bmj.m1985 32444460 PMC7243036

[3] Stokes EK, Zambrano LD, Anderson KN, Marder EP, Raz KM, El Burai Felix S, Tie Y, Fullerton KE. Coronavirus Disease 2019 Case Surveillance - United States, January 22-May 30, 2020. MMWR Morb Mortal Wkly Rep. 2020; 69:759–65. 10.15585/mmwr.mm6924e2 32555134 PMC7302472

[4] Mahmud SMH, Al-Mustanjid M, Akter F, Rahman MS, Ahmed K, Rahman MH, Chen W, Moni MA. Bioinformatics and system biology approach to identify the influences of SARS-CoV-2 infections to idiopathic pulmonary fibrosis and chronic obstructive pulmonary disease patients. Brief Bioinform. 2021; 22:bbab115. 10.1093/bib/bbab115 33847347 PMC8083324

[5] Taz TA, Ahmed K, Paul BK, Kawsar M, Aktar N, Mahmud SMH, Moni MA. Network-based identification genetic effect of SARS-CoV-2 infections to Idiopathic pulmonary fibrosis (IPF) patients. Brief Bioinform. 2021; 22:1254–66. 10.1093/bib/bbaa235 33024988 PMC7665362

[6] Libruder C, Hershkovitz Y, Ben-Yaish S, Tanne D, Keinan-Boker L, Binyaminy B. An increased risk for ischemic stroke in the short-term period following COVID-19 infection: A nationwide population-based study. Neuroepidemiology. 2023. [Epub ahead of print]. 10.1159/000531163 37399799 PMC11251667

[7] Toscano G, Palmerini F, Ravaglia S, Ruiz L, Invernizzi P, Cuzzoni MG, Franciotta D, Baldanti F, Daturi R, Postorino P, Cavallini A, Micieli G. Guillain-Barré Syndrome Associated with SARS-CoV-2. N Engl J Med. 2020; 382:2574–6. 10.1056/NEJMc2009191 32302082 PMC7182017

[8] Cen G, Liu L, Wang J, Wang X, Chen S, Song Y, Liang Z. Weighted Gene Co-Expression Network Analysis to Identify Potential Biological Processes and Key Genes in COVID-19-Related Stroke. Oxid Med Cell Longev. 2022; 2022:4526022. 10.1155/2022/4526022 35557984 PMC9088964

[9] Li W, Li W, Zhang W, Wang H, Yu L, Yang P, Qin Y, Gan M, Yang X, Huang L, Hao Y, Geng D. Exogenous melatonin ameliorates steroid-induced osteonecrosis of the femoral head by modulating ferroptosis through GDF15-mediated signaling. Stem Cell Res Ther. 2023; 14:171. 10.1186/s13287-023-03371-y 37400902 PMC10318673

[10] Zhang R, Sun C, Chen X, Han Y, Zang W, Jiang C, Wang J, Wang J. COVID-19-Related Brain Injury: The Potential Role of Ferroptosis. J Inflamm Res. 2022; 15:2181–98. 10.2147/JIR.S353467 35411172 PMC8994634

[11] Pomilio AB, Vitale AA, Lazarowski AJ. COVID-19 and Alzheimer's Disease: Neuroinflammation, Oxidative Stress, Ferroptosis, and Mechanisms Involved. Curr Med Chem. 2023; 30:3993–4031. 10.2174/0929867329666221003101548 36200215

[12] Chen X, Kang R, Kroemer G, Tang D. Ferroptosis in infection, inflammation, and immunity. J Exp Med. 2021; 218:e20210518. 10.1084/jem.20210518 33978684 PMC8126980

[13] Ren X, Wen W, Fan X, Hou W, Su B, Cai P, Li J, Liu Y, Tang F, Zhang F, Yang Y, He J, Ma W, et al. COVID-19 immune features revealed by a large-scale single-cell transcriptome atlas. Cell. 2021; 184:1895–913.e19. 10.1016/j.cell.2021.01.053 33657410 PMC7857060

[14] Lévy Y, Wiedemann A, Hejblum BP, Durand M, Lefebvre C, Surénaud M, Lacabaratz C, Perreau M, Foucat E, Déchenaud M, Tisserand P, Blengio F, Hivert B, et al, and French COVID cohort study group. CD177, a specific marker of neutrophil activation, is associated with coronavirus disease 2019 severity and death. iScience. 2021; 24:102711. 10.1016/j.isci.2021.102711 34127958 PMC8189740

[15] O'Connell GC, Treadway MB, Petrone AB, Tennant CS, Lucke-Wold N, Chantler PD, Barr TL. Peripheral blood AKAP7 expression as an early marker for lymphocyte-mediated post-stroke blood brain barrier disruption. Sci Rep. 2017; 7:1172. 10.1038/s41598-017-01178-5 28446746 PMC5430856

[16] Overmyer KA, Shishkova E, Miller IJ, Balnis J, Bernstein MN, Peters-Clarke TM, Meyer JG, Quan Q, Muehlbauer LK, Trujillo EA, He Y, Chopra A, Chieng HC, et al. Large-Scale Multi-omic Analysis of COVID-19 Severity. Cell Syst. 2021; 12:23–40.e7. 10.1016/j.cels.2020.10.003 33096026 PMC7543711

[17] Krug T, Gabriel JP, Taipa R, Fonseca BV, Domingues-Montanari S, Fernandez-Cadenas I, Manso H, Gouveia LO, Sobral J, Albergaria I, Gaspar G, Jiménez-Conde J, Rabionet R, et al. TTC7B emerges as a novel risk factor for ischemic stroke through the convergence of several genome-wide approaches. J Cereb Blood Flow Metab. 2012; 32:1061–72. 10.1038/jcbfm.2012.24 22453632 PMC3367223

[18] Chong W, Shang L, Liu J, Fang Z, Du F, Wu H, Liu Y, Wang Z, Chen Y, Jia S, Chen L, Li L, Chen H. m^6^A regulator-based methylation modification patterns characterized by distinct tumor microenvironment immune profiles in colon cancer. Theranostics. 2021; 11:2201–17. 10.7150/thno.52717 33500720 PMC7797678

[19] Newman AM, Liu CL, Green MR, Gentles AJ, Feng W, Xu Y, Hoang CD, Diehn M, Alizadeh AA. Robust enumeration of cell subsets from tissue expression profiles. Nat Methods. 2015; 12:453–7. 10.1038/nmeth.3337 25822800 PMC4739640

[20] Zhou N, Bao J. FerrDb: a manually curated resource for regulators and markers of ferroptosis and ferroptosis-disease associations. Database (Oxford). 2020; 2020:baaa021. 10.1093/database/baaa021 32219413 PMC7100629

[21] Verveen A, Verfaillie SCJ, Visser D, Csorba I, Coomans EM, Koch DW, Appelman B, Barkhof F, Boellaard R, de Bree G, van de Giessen EM, Golla S, van Heugten CM, et al. Neurobiological basis and risk factors of persistent fatigue and concentration problems after COVID-19: study protocol for a prospective case-control study (VeCosCO). BMJ Open. 2023; 13:e072611. 10.1136/bmjopen-2023-072611 37399444 PMC10314688

[22] Bulla R, Rossi L, Furlanis G, Agostinis C, Toffoli M, Balduit A, Mangogna A, Liccari M, Morosini G, Kishore U, Manganotti P. A likely association between low mannan-binding lectin level and brain fog onset in long COVID patients. Front Immunol. 2023; 14:1191083. 10.3389/fimmu.2023.1191083 37398656 PMC10312368

[23] Lindqvist I, Cunningham JL, Mulder J, Feresiadou A, Rostami E, Virhammar J, Kumlien E. Myoclonus in patients with COVID-19: Findings of autoantibodies against brain structures in cerebrospinal fluid. Eur J Neurol. 2023. [Epub ahead of print]. 10.1111/ene.15958 37392418

[24] Zhang L, Ren J, Shi P, Lu D, Zhao C, Su Y, Zhang L, Huang J. The Immunological Regulation Roles of Porcine β-1, 4 Galactosyltransferase V (B4GALT5) in PRRSV Infection. Front Cell Infect Microbiol. 2018; 8:48. 10.3389/fcimb.2018.00048 29546034 PMC5837993

[25] Jackson RM, Griesel BA, Short KR, Sparling D, Freeman WM, Olson AL. Weight Loss Results in Increased Expression of Anti-Inflammatory Protein CRISPLD2 in Mouse Adipose Tissue. Obesity (Silver Spring). 2019; 27:2025–36. 10.1002/oby.22652 31746554 PMC6873817

[26] Zhang S, Pei L, Qu J, Sun L, Jiang W, Li W, Lin Z, Chen D. CRISPLD2 attenuates pro-inflammatory cytokines production in HMGB1-stimulated monocytes and septic mice. Am J Transl Res. 2021; 13:4080–91. 34150000 PMC8205833

[27] Liu Y, Liao XW, Qin YZ, Mo XW, Luo SS. Identification of *F5* as a Prognostic Biomarker in Patients with Gastric Cancer. Biomed Res Int. 2020; 2020:9280841. 10.1155/2020/9280841 32190689 PMC7064826

[28] Zhang HL, Hu BX, Li ZL, Du T, Shan JL, Ye ZP, Peng XD, Li X, Huang Y, Zhu XY, Chen YH, Feng GK, Yang D, et al. PKCβII phosphorylates ACSL4 to amplify lipid peroxidation to induce ferroptosis. Nat Cell Biol. 2022; 24:88–98. 10.1038/s41556-021-00818-3 35027735

[29] Liao P, Wang W, Wang W, Kryczek I, Li X, Bian Y, Sell A, Wei S, Grove S, Johnson JK, Kennedy PD, Gijón M, Shah YM, Zou W. CD8^+^ T cells and fatty acids orchestrate tumor ferroptosis and immunity via ACSL4. Cancer Cell. 2022; 40:365–78.e6. 10.1016/j.ccell.2022.02.003 35216678 PMC9007863

[30] Qiu Y, Cao Y, Cao W, Jia Y, Lu N. The Application of Ferroptosis in Diseases. Pharmacol Res. 2020; 159:104919. 10.1016/j.phrs.2020.104919 32464324

[31] Sun Y, Chen P, Zhai B, Zhang M, Xiang Y, Fang J, Xu S, Gao Y, Chen X, Sui X, Li G. The emerging role of ferroptosis in inflammation. Biomed Pharmacother. 2020; 127:110108. 10.1016/j.biopha.2020.110108 32234642

[32] Xia H, Zhang Z, You F. Inhibiting ACSL1-Related Ferroptosis Restrains Murine Coronavirus Infection. Viruses. 2021; 13:2383. 10.3390/v13122383 34960652 PMC8708337

[33] Nomura N, Zu YL, Maekawa T, Tabata S, Akiyama T, Ishii S. Isolation and characterization of a novel member of the gene family encoding the cAMP response element-binding protein CRE-BP1. J Biol Chem. 1993; 268:4259–66. 8440710

[34] Conkright MD, Montminy M. CREB: the unindicted cancer co-conspirator. Trends Cell Biol. 2005; 15:457–9. 10.1016/j.tcb.2005.07.007 16084096

[35] Impey S, McCorkle SR, Cha-Molstad H, Dwyer JM, Yochum GS, Boss JM, McWeeney S, Dunn JJ, Mandel G, Goodman RH. Defining the CREB regulon: a genome-wide analysis of transcription factor regulatory regions. Cell. 2004; 119:1041–54. 10.1016/j.cell.2004.10.032 15620361

[36] Wang S, Qiu J, Liu L, Su C, Qi L, Huang C, Chen X, Zhang Y, Ye Y, Ding Y, Liang L, Liao W. CREB5 promotes invasiveness and metastasis in colorectal cancer by directly activating MET. J Exp Clin Cancer Res. 2020; 39:168. 10.1186/s13046-020-01673-0 32843066 PMC7446182

[37] Wu YH, Yeh IJ, Phan NN, Yen MC, Hung JH, Chiao CC, Chen CF, Sun Z, Hsu HP, Wang CY, Lai MD. Gene signatures and potential therapeutic targets of Middle East respiratory syndrome coronavirus (MERS-CoV)-infected human lung adenocarcinoma epithelial cells. J Microbiol Immunol Infect. 2021; 54:845–57. 10.1016/j.jmii.2021.03.007 34176764 PMC7997684

[38] Kratofil RM, Kubes P, Deniset JF. Monocyte Conversion During Inflammation and Injury. Arterioscler Thromb Vasc Biol. 2017; 37:35–42. 10.1161/ATVBAHA.116.308198 27765768

[39] Knoll R, Schultze JL, Schulte-Schrepping J. Monocytes and Macrophages in COVID-19. Front Immunol. 2021; 12:720109. 10.3389/fimmu.2021.720109 34367190 PMC8335157

[40] Merad M, Martin JC. Pathological inflammation in patients with COVID-19: a key role for monocytes and macrophages. Nat Rev Immunol. 2020; 20:355–62. 10.1038/s41577-020-0331-4 32376901 PMC7201395

[41] Witkowski M, Tizian C, Ferreira-Gomes M, Niemeyer D, Jones TC, Heinrich F, Frischbutter S, Angermair S, Hohnstein T, Mattiola I, Nawrath P, McEwen S, Zocche S, et al. Untimely TGFβ responses in COVID-19 limit antiviral functions of NK cells. Nature. 2021; 600:295–301. 10.1038/s41586-021-04142-6 34695836

[42] Cox RJ, Brokstad KA. Not just antibodies: B cells and T cells mediate immunity to COVID-19. Nat Rev Immunol. 2020; 20:581–2. 10.1038/s41577-020-00436-4 32839569 PMC7443809

[43] Sette A, Crotty S. Adaptive immunity to SARS-CoV-2 and COVID-19. Cell. 2021; 184:861–80. 10.1016/j.cell.2021.01.007 33497610 PMC7803150

