# Supplementary material for: Single-cell RNA sequencing and multiple bioinformatics methods to identify the immunity and ferroptosis-related biomarkers of SARS-CoV-2 infections to ischemic stroke
Source: Aging (Albany NY). 2023 Aug 21;15(16):8237–57. doi: 10.18632/aging.204966 (PMC10497002; doi:10.18632/aging.204966)
Supplement: Supplementary Figures [file aging-15-204966-s001.pdf]

SUPPLEMENTARY FIGURES

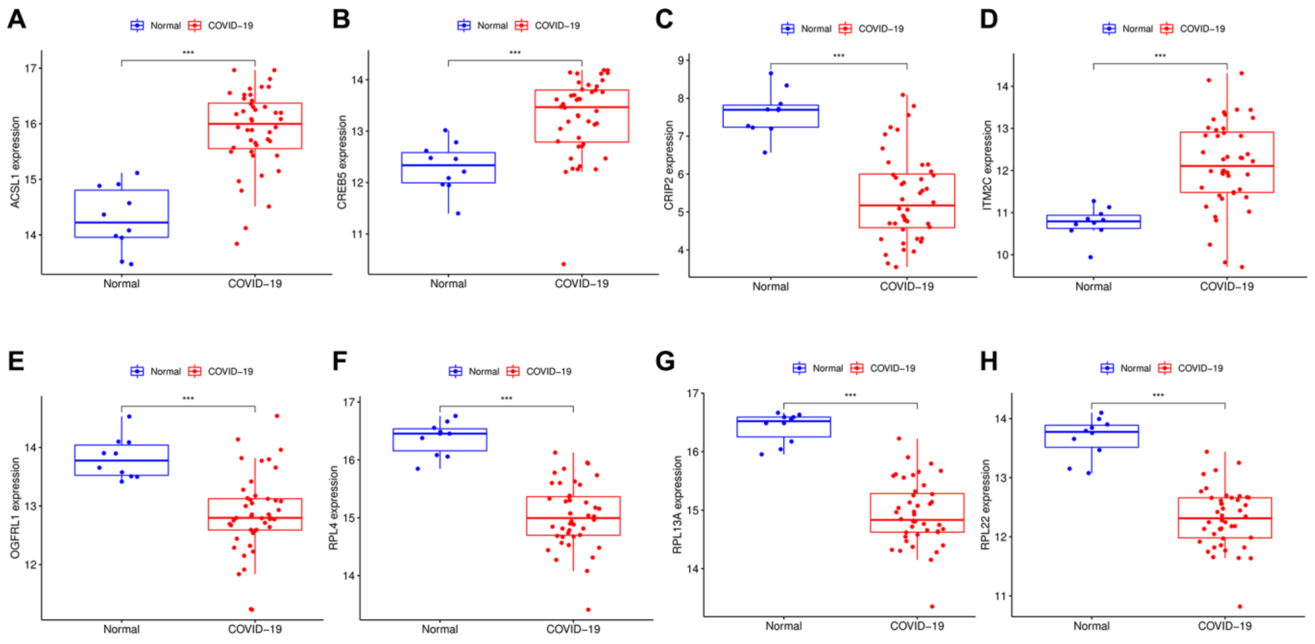

Supplementary Figure 1. Expression of hub genes in COVID-19 train cohort GSE171110. (**A**) The expression of ACSL1 in GSE171110. (**B**) The expression of CREB5 in GSE171110. (**C**) The expression of CRIP2 in GSE171110. (**D**) The expression of ITM2C in GSE171110. (**E**) The expression of OGFRL1 in GSE171110. (**F**) The expression of RPL4 in GSE171110. (**G**) The expression of RPL13A in GSE171110. (**H**) The expression of RPL22 in GSE171110.

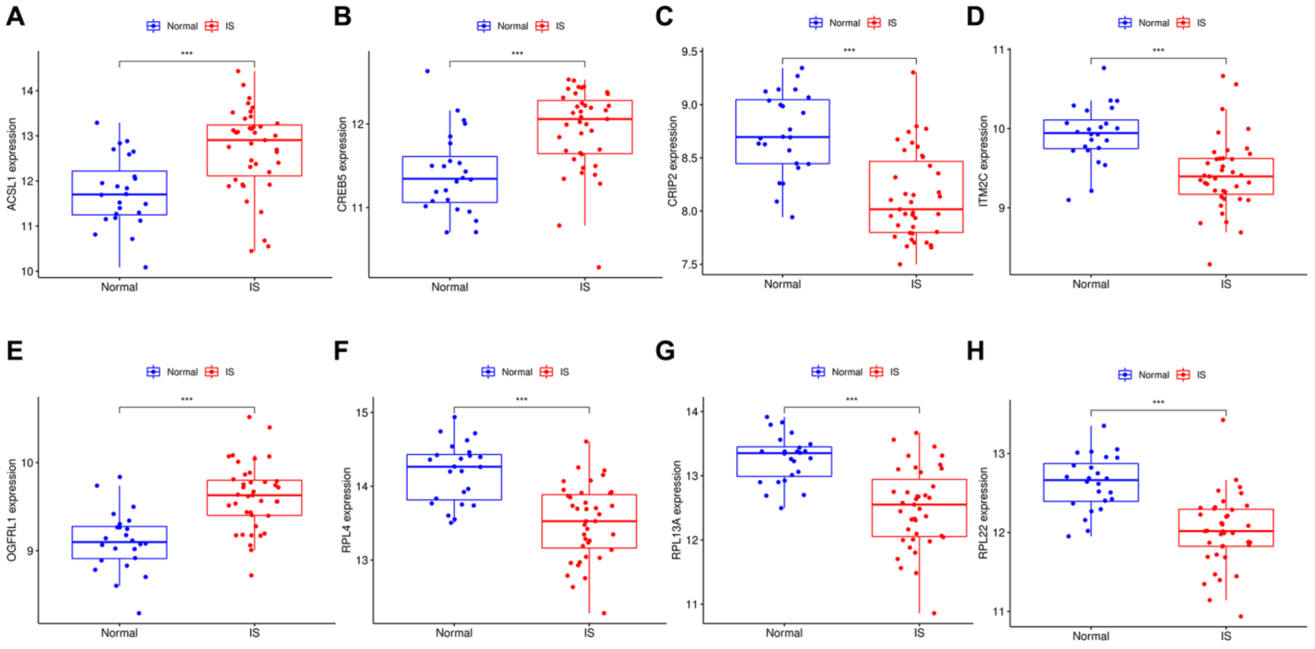

Supplementary Figure 2. Expression of hub genes in IS train cohort GSE16561. (**A**) The expression of ACSL1 in GSE16561. (**B**) The expression of CREB5 in GSE16561. (**C**) The expression of CRIP2 in GSE16561. (**D**) The expression of ITM2C in GSE16561. (**E**) The expression of OGFRL1 in GSE16561. (**F**) The expression of RPL4 in GSE16561. (**G**) The expression of RPL13A in GSE16561. (**H**) The expression of RPL22 in GSE16561.

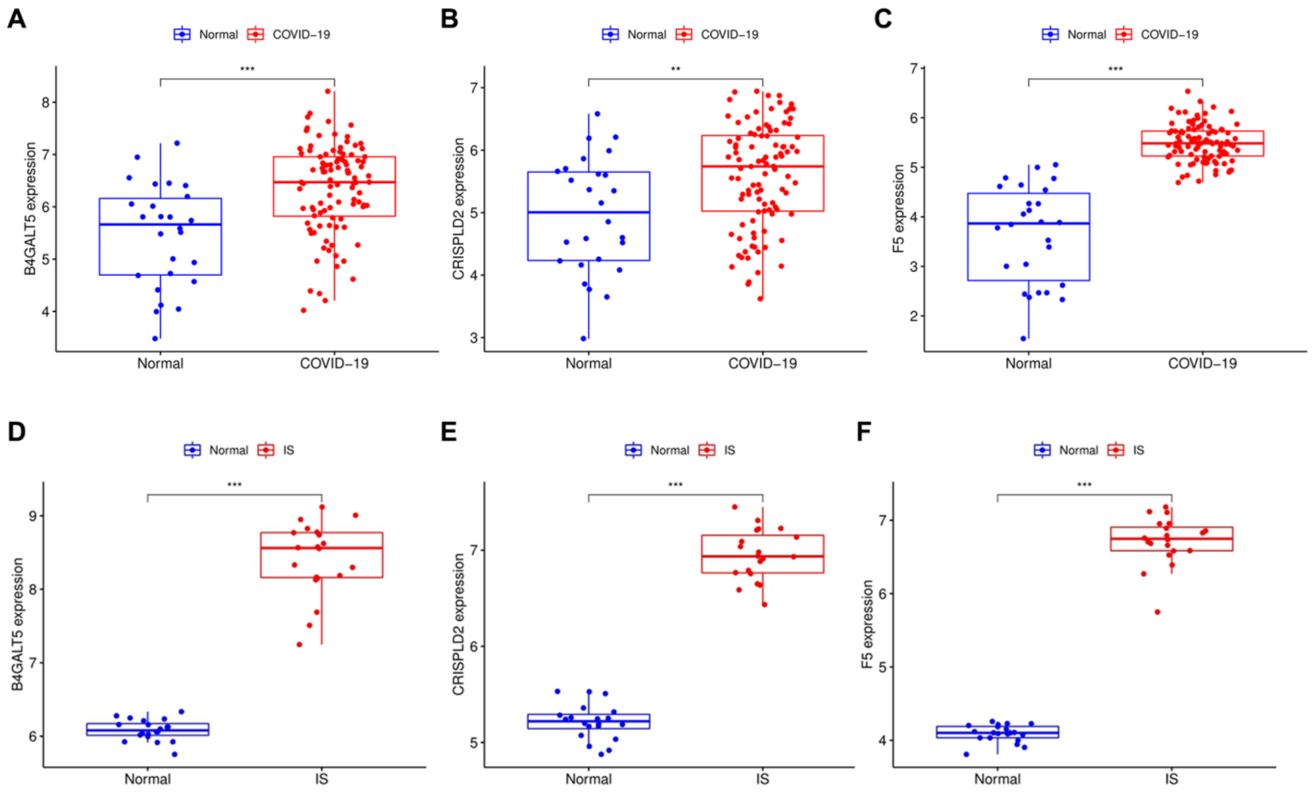

Supplementary Figure 3. Expression of hub genes in COVID-19 validation cohort GSE157103 and IS validation cohort GSE22255. (**A**) The expression of B4GALT5 in GSE157103. (**B**) The expression of CRISPLD2 in GSE157103. (**C**) The expression of F5 in GSE157103. (**D**) The expression of B4GALT5 in GSE22255. (**E**) The expression of CRISPLD2 in GSE22255. (**F**) The expression of F5 in GSE22255.

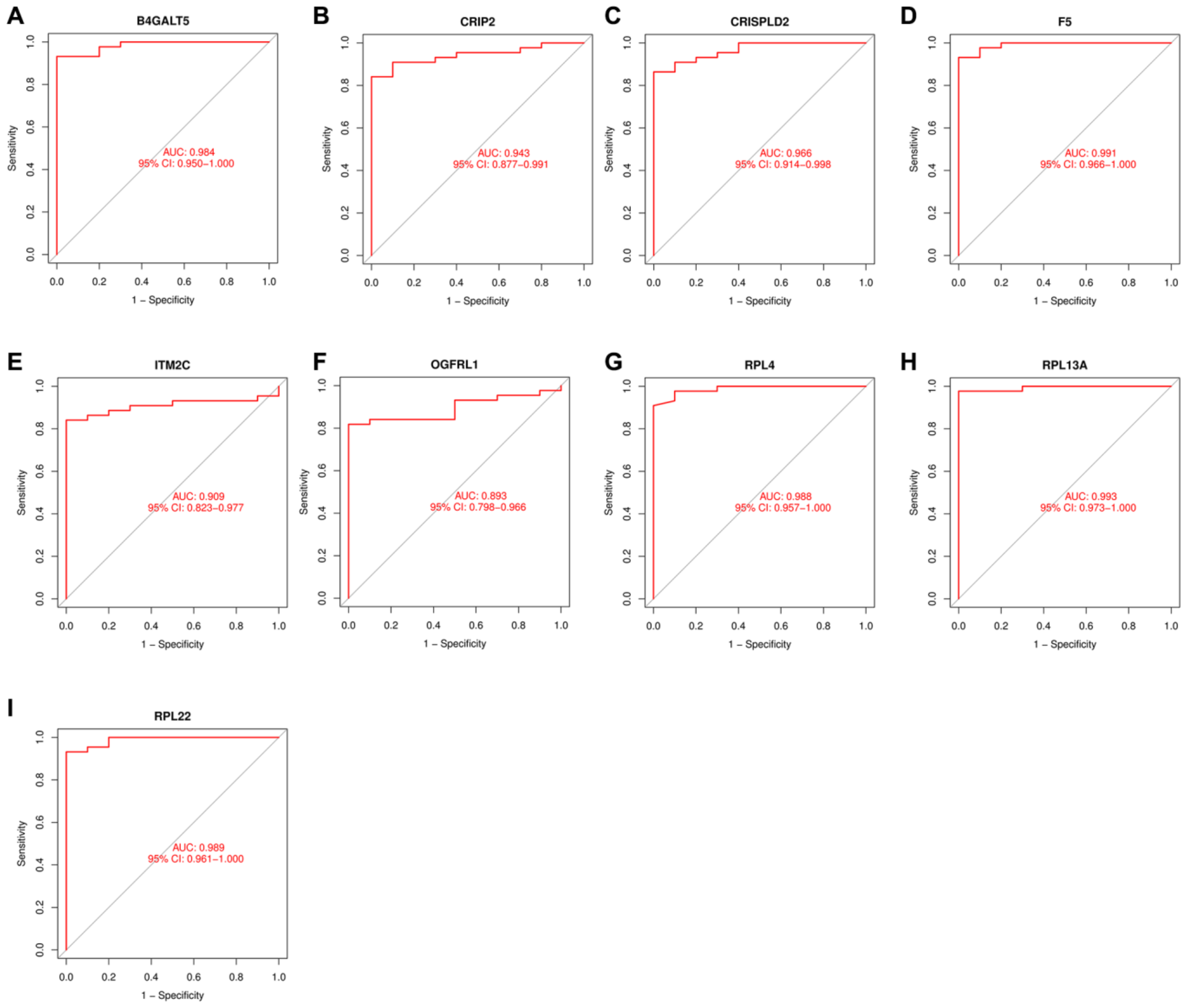

Supplementary Figure 4. ROC curves for hub genes in COVID-19 train cohort GSE171110. (**A**) The ROC curve of B4GALT5 in GSE171110. (**B**) The ROC curve of CRIP2 in GSE171110. (**C**) The ROC curve of CRISPLD2 in GSE171110. (**D**) The ROC curve of F5 in GSE171110. (**E**) The ROC curve of ITM2C in GSE171110. (**F**) The ROC curve of OGFRL1 in GSE171110. (**G**) The ROC curve of RPL4 in GSE171110. (**H**) The ROC curve of RPL13A in GSE171110. (**I**) The ROC curve of RPL22 in GSE171110.

Rank	Pert_iname	Moa	Target name	Fdr_q_nlog10	Norm_CS
1	ARRY-334543	EGFR inhibitor	ERBB2|EGFR|ERBB4	15.6536	-1.9725
2	BIX-02189	MEK inhibitor	MAP2K5|MAPK7	15.6536	-1.9445
3	etoposide	Topoisomerase inhibitor	TOP2A|TOP2B|CYP2E1|CYP3A5	15.6536	-1.9418
4	SB-939	HDAC inhibitor	HDAC1|HDAC2|HDAC10|HDAC3|HDAC4|HDAC5|HDAC9	15.6536	-1.9247
5	cabazitaxel	Tubulin inhibitor	CYP2C8|CYP3A5|SLCO1B3|TUBA4A|TUBB|TUBB1	15.6536	-1.9246
6	amsacrine	Topoisomerase inhibitor	TOP2A|KCNH2	15.6536	-1.906
7	amsacrine	Topoisomerase inhibitor	TOP2A|KCNH2	15.6536	-1.9
8	docetaxel	Tubulin inhibitor	TUBB|BCL2|MAP2|MAP4|MAPT|NR1I2|TUBB1	15.6536	-1.8952
9	teniposide	Topoisomerase inhibitor	TOP2A|CYP3A5|TOP2B	15.6536	-1.8803
10	palbociclib	CDK inhibitor	CDK4|CDK6|CCND3	15.6536	-1.8635

Supplementary Figure 5. Potential drugs for COVID-19 predicted by CMAP database.

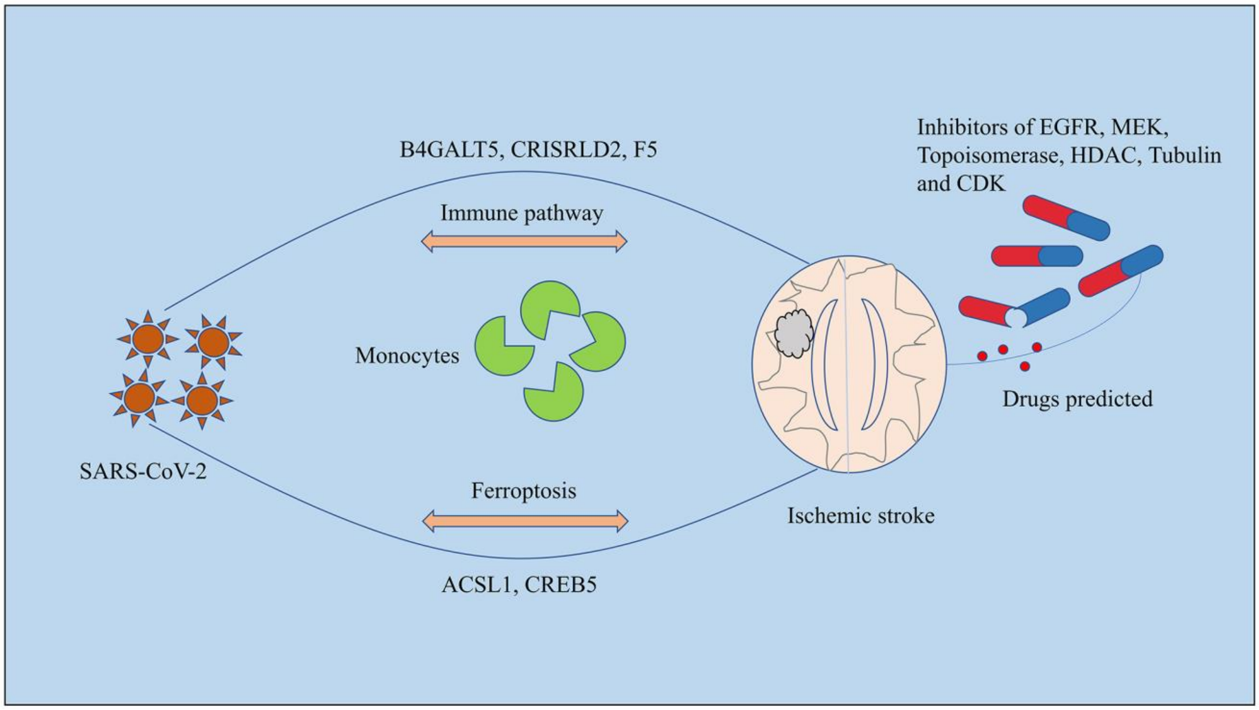

Supplementary Figure 6. Schematic diagram of the research.
